# Awe/Gratitude as an Experiential Aspect of Spirituality and Its Association to Perceived Positive Changes During the COVID-19 Pandemic

**DOI:** 10.3389/fpsyt.2021.642716

**Published:** 2021-04-20

**Authors:** Arndt Büssing, Daniela Rodrigues Recchia, Thomas Dienberg, Janusz Surzykiewicz, Klaus Baumann

**Affiliations:** ^1^Professorship Quality of Life, Spirituality and Coping, Faculty of Health, Witten/Herdecke University, Herdecke, Germany; ^2^IUNCTUS - Competence Center for Christian Spirituality, Philosophical-Theological Academy, Münster, Germany; ^3^Chair of Research Methods and Statistics in Psychology, Faculty of Health, Witten/Herdecke University, Witten, Germany; ^4^Chair of Social Pedagogy, Catholic University Eichstätt-Ingolstadt, Eichstätt, Germany; ^5^Cardinal Wyszynski University, Faculty of Education, Warsaw, Poland; ^6^Caritas Science and Christian Social Work, Faculty of Theology, Albert-Ludwig-University, Freiburg, Germany

**Keywords:** awe, gratitude, spirituality, resilience, burden, perceived changes, COVID-19 pandemic

## Abstract

**Background:** While the COVID-19 pandemic has affected the lives of almost all people worldwide, many people observed also positive changes in their attitudes and behaviors. This can be seen in the context of posttraumatic growth. These perceived changes refer to five main categories: Nature/Silence/Contemplation, Spirituality, Relationships, Reflection on life, and Digital media usage. A previous study with persons recruited in June 2020 directly after the lockdown in Germany showed that the best predictors of these perceived changes related to the Corona pandemic were the ability to mindfully stop and pause in distinct situations, to be “spellbound at the moment” and to become “quiet and devout,” indicating moments of wondering awe, with subsequent feelings of gratitude. Now, we intended to analyze (1) by whom and how strongly awe/gratitude was experienced during the COVID-19 pandemic, and (2) how these feelings relate to perceived changes and experienced burden, and (3) whether or not feelings of awe/gratitude contribute to participants' well-being or may buffer perceived burden in terms of a resilience factor.

**Methods:** Online survey with standardized questionnaires [i.e., WHO-Five Well-being Index (WHO5), Life satisfaction (BMLSS), Awe/Gratitude scale (GrAw-7), and Perceived Changes Questionnaire (PCQ)] among 2,573 participants (68% women; mean age 48.7 ± 14.2 years, 74% with a Christian affiliation) from Germany recruited between June and November 2020.

**Results:** Awe/Gratitude scored significantly higher particularly among women (Cohen's *d* = 0.40), older persons (*d* = 0.88), persons who rely on their faith as a “stronghold in difficult times” (*d* = 0.99), those with higher well-being (*d* = 0.70), and lower perceptions of loneliness (*d* = 0.49). With respect to perceived changes during the pandemic, more intense feelings of Awe/Gratitude were particularly related to Nature/Silence/Contemplation (*r* = 0.41), Spirituality (*r* = 0.41), and Relationships (*r* = 0.33). Regression analyses revealed that the best predictors of Awe/Gratitude (*R*^2^ = 0.40) were the frequency of meditation, female gender, life satisfaction and well-being, faith as a stronghold, and perceived burden and also life reflection, while Nature/Silence/Contemplation and Relationships had a further, but weaker, impact on Awe/Gratitude as a dependent variable. Awe/Gratitude was moderately associated with well-being (*r* = 0.32) and would predict 9% of participants' well-being variance. The best predictors of participants' well-being were multidimensional life satisfaction and low perceived burden (related to the pandemic), and further Awe/Gratitude and Nature/Silence/Contemplation; these would explain 47% of variance in well-being scores. However, Awe/Gratitude cannot be regarded as a buffer of the negative aspects of the COVID-19 pandemic, as it is only marginally (though negatively) related to perceived burden (*r* = −0.15). Mediation analysis showed that Awe/Gratitude mediates 42% of the link between well-being as a predictor on Nature/Silence/Contemplation as an outcome and has a direct effect of β = 0.15 (*p* < 0.001) and an indirect effect of β = 0.11 (*p* < 0.001). Further, Awe/Gratitude mediates 38% (*p* < 0.001) of the link between Nature/Silence/Contemplation as a predictor on well-being as the outcome; the direct effect is β = 0.18 (*p* < 0.001), and the indirect effect is β = 0.11 (*p* < 0.001).

**Conclusions:** The general ability to experience Awe/Gratitude particularly during the COVID-19 pandemic may sensitize to perceive the world around (including nature and concrete persons) more intensely, probably in terms of, or similar to, posttraumatic growth. As this awareness toward specific moments and situations that deeply “touch” a person was higher in persons with more intense meditation or prayer practice, one may assume that these practices may facilitate these perceptions in terms of a training. However, the experience of Awe/Gratitude does not necessarily buffer against adverse events in life and cannot prevent perceived burden due to the corona pandemic, but it facilitates to, nevertheless, perceive positive aspects of life even within difficult times. As Awe/Gratitude is further mediating the effects of Nature/Silence/Contemplation on well-being, intervention programs could help to train these perceptions, as these self-transcendent feelings are also related to prosocial behaviors with respectful treatment of others and commitment to persons in needs, and well-being.

## Introduction

The COVID-19 pandemic has changed the lives of almost all people worldwide in one way or the other. Many infected persons have died, others have recovered but have to cope with chronic health affections, and others recovered without relevant restrictions; a lot of people have lost their jobs, or are working in short-time, while others are in home-office or continue at their workplace. Many persons at risk avoid social contacts and feel isolated (1, 2), while others do not care too much about social restrictions (3), or even discuss why face-mask usage might be harmful (4). There are, in fact, heterogeneous ways to cope with the implications of the pandemic (5). A recent systematic review underlines that in the general population, there is a high prevalence of stress, anxiety, and depression because of the COVID-19 pandemic (6). Clinicians meet patients in depressive states and others with signs of “defeat stress” (7). Tumor patients, as an example of persons at risk, reported to be in fear of being infected, of having and complicated courses of disease and of staying helpless; they were irritated about conflicting information about the danger and about the course of the COVID-19 infection in the media (1, 2).

Nevertheless, related to the “extra time” at home during the lockdown, many people not only had fears and worries; they also perceived positive changes in their attitudes and behaviors. Tumor patients, for example, perceived nature and silence and also their relationships more intensely, some had a stronger interest in spiritual issues, while others perceived loneliness and ruminated worrying reflections (2). In a survey among a more general population from Germany, these perceived changes were operationalized and measured in terms of (1) *Nature/Silence/Contemplation*, (2) *Spirituality*, (3) *Relationships*, (4) *Reflection on life*, and (5) *Digital media use* (8). Strongest changes were observed for *Relationships* and *Nature/Silence/Contemplation*. All perceived changes were stronger among older persons, among those with higher well-being, and those who relied on their faith as a resource. Interestingly, best predictors of these perceived changes were the ability to mindfully stop and pause in distinct situations, to be “spellbound at the moment” and to become “quiet and devout,” indicating moments of wondering awe, with subsequent feelings of gratefulness (8).

Perceptions of wondering awe, which can be considered as a variety of religious experience (9) can also be interpreted as an experiential aspect of “secular spirituality” as no specific religious belief is required and thus can be perceived also by non-religious persons (10, 11). Awe in terms of admiration (as a weaker experience) or even of overwhelming astonishment (as a stronger experience) is an emotional perception triggered by “outstanding” experiences, situations, nature, music, and persons (12–14). Keltner and Haidt (12) assumed that perceptions of awe might be experienced also in “times of tremendous social change;” in consequence, this perception is probably relevant particularly in times of the COVID-19 pandemic, too.

In line with the findings of Yaden et al. (15), qualitative analyses revealed that the range of awe triggers is wide, encompassing, e.g., Experience of nature, Perceiving the sacred in creation, Encounter with impressive people, Birth/own children, Accompanying the dying, Professional situations/Crises, Times of silence, Art and Music, Sacred buildings, and Special spiritual practices/ceremonies (Büssing et al., in preparation). Depending on the triggers, the resulting emotions can be overwhelming, while smaller moments of wonder (or admiration) are more common. The readiness to be touched and to mindfully perceive such moments seems to be higher in religious persons compared with non-religious/non-spiritual persons (10, 11). As Awe/Gratitude is related to the frequency of spiritual practices (i.e., meditation and praying), these practices can be considered as sensitizers for mindful encounters with nature, others, and with whatever a person may regard as sacred (10). Praying and meditation seem to facilitate such perceptions of connectedness with nature and others. Such a sense of connection and belonging was observed by Prade and Saraougly (16), too.

In Franciscan brothers and sisters, who have committed themselves to live a devoted life, which may facilitate such feelings, Awe/Gratitude was related to their intention to seek God in silence and prayer, with peaceful attitudes and respectful treatment of others and nature, and special care for persons in need (17). In terms of an “inner transformation,” these perceptions may have consequences for a person's life, as they may change their behaviors and attitudes (12, 17). Self-transcendent emotions may also intensify a person's spirituality and their religious and spiritual feelings (18). Further, persons with a gratefulness disposition seem to be more able to perceive these feelings (10), and even persons with depressive diseases can experience them (19).

At any rate, Awe/Gratitude as a construct of spiritual awareness can be regarded as an additional dimension of a persons' quality of life. However, Awe/Gratitude is weakly associated with well-being and marginally only correlated to multidimensional life satisfaction (17). When one perceives awe in terms of the Sacred (God), then one may also feel more connected with others and may be more satisfied with life (20). In addition, gratitude as a life orientation is associated with well-being and positive social relationships (21), and with lower risk of major depression, generalized anxiety disorder, phobia, and drug abuse (22), as it may help to reframe the negative emotions (23). For religious persons, gratitude is also related with perceived closeness to God and to a secure attachment to God (24). Furthermore, spiritual transcendence and connectedness with transcendent sources of meaning outside is higher in grateful persons (25).

On the other hand, therefore, well-being is not necessarily a prerequisite to perceive Awe/Gratitude. Feelings of Awe/Gratitude may arise because of a more open and mindful awareness for those things in life that are of value and thus can emotionally touch a person—in spite of difficulties in life (i.e., coping with the outcomes of the COVID-19 pandemic). Such an awareness shift has been frequently observed in persons who undergo difficult life situations or phases of illness (26). In this perspective, one can even relate the outcomes of these experiences to the concept of posttraumatic growth (27, 28) or of spiritual transformation (29, 30), where people change their attitudes and make new resolutions, become (at least for some time) more conscious, more attentive, more mindful, more spiritually minded, etc.

As perceptions of awe and gratitude were among the best predictors of persons' perceived changes due to the Corona pandemic (2), we intended to analyze whether Awe/Gratitude could be regarded as a resilience factor to cope with the impacts of the pandemic on their lives. Theoretical considerations drawn from experimental studies would indicate that awe may buffer negative feelings (31, 32). Also in an experimental group of people waiting for the results of an intelligence test or peer feedback, positive emotions and less anxiety were observed when they experienced “awe conditions” compared with neutral conditions, and these findings were independent from a person's predisposition to experience awe (33). As positive emotions usually help to adapt in difficult situations, it was suggested that also gratitude could be a resilience factor [(34); cf. (35)]. We therefore intended to analyze (1) how strongly and by whom Awe/Gratitude was perceived during the corona pandemic, and (2) how these feelings relate to perceived changes and experienced burden during the pandemic, and (3) whether or not feelings of awe/gratitude contribute to participants' well-being or may buffer perceived burden.

## Methods

### Recruitment of Participants

Participants were recruited within 6 months (from June 9 to November 30, 2020). The snowball sampling started in different networks in Germany, i.e., university students and staff, research collaborators, religious orders and church communities, Rotary Club members, Facebook sites, diocesan websites, etc. All contacted persons and networks were invited to share the information and link where possible. Within this time frame, we were able to include persons from the first wave of the corona pandemic, from the “relaxation” time in summer, and in the meantime from the second wave of fall and winter 2020.

Participants were assured confidentially and were informed about the purpose of the study and data protection information at the starting page of the online survey. By filling in the anonymous questionnaire, interested persons consented to participate. Neither concrete identification of personal details nor IP addresses were recorded to realize and guarantee full anonymity. Therefore, we were unable to control for multiple entries.

#### Awe and Gratitude

To address times of pausing for “wonder” (Awe) in specific situations as a perceptive aspect of spirituality, we used the seven-item Awe/Gratitude scale (GrAw-7) (10). This single-factor scale has good psychometric properties (Cronbach's alpha = 0.82) and uses items such as “In certain places, I become very quiet and devout,” “I stop and am captivated by the beauty of nature,” “I pause and stay spellbound at the moment,” “I stop and then think of so many things for which I'm really grateful.” The scale thus addresses a person's emotional reaction toward an immediate and “captive” experience. All items were scored on a four-point scale (0—*never*; 1—*seldom*; 2—*often*; 3—*regularly*), referred to as a 100-point scale. In this sample, Cronbach's alpha is 0.87, and the single-factor structure was confirmed (explaining 57% of variance).

#### Perception of Changes

Perceived changes due to the Corona pandemic were measured with the 24-item Perceived Changes Questionnaire (PCQ) (8). This newly developed instrument differentiates five dimensions (factors) with good internal consistence: (1) *Nature/Silence/Contemplation* (Cronbach's alpha = 0.87), (2) *Spirituality* (Cronbach's alpha = 0.83), (3) *Relationships* (Cronbach's alpha = 0.80), (4) (worrying) *Reflection on life* (Cronbach's alpha = 0.74), (5) *Digital media usage* (Cronbach's alpha = 0.74). The respective items refer to perceptions that were reported by various persons at the start of the COVID-19-related lockdown. The respective items were introduced by the phrase “Due to the current situation…,” (referring to the Corona pandemic) and scored on a five-point agreement scale (0—*does not apply at all*; 1—*does not truly apply*; 2—*neither yes nor no*; 3—*applies quite a bit*; 4—*applies very much*). Specific items are “I perceive the relationship with my partner/family more intensely,” “I pay more attention to what's really important in life,” “I perceive nature more intensely,” “I enjoy quiet times of reflection,” “I am connected to friends via digital media,” “I deal more with spiritual/religious questions,” “I pray/meditate more than before,” “I'm more concerned about the lifetime that I have,” etc.

#### Well-Being Index

Participants' well-being (within the last 2 weeks) was measured with the WHO-Five Well-being Index (WHO-5) (36). It uses items such as “I have felt cheerful and in good spirits” or “My daily life has been filled with things that interest me.” The frequency of these experiences is scored from *at no time* (0) to *all of the times* (5). Here, we report the sum scores ranging from 0 to 25 and also the 100% level scores ranging from 0 to 100. Scores <13 (or <50) would indicate reduced well-being or even depressive states. In this sample, Cronbach's alpha = 0.89.

#### Life Satisfaction

Life satisfaction was measured with the Brief Multidimensional Life Satisfaction Scale (BMLSS) (37). It covers five main topics: intrinsic (oneself and life in general), social (friendships and family life), external (work situation and where one live), prospective dimensions (financial situation and future prospects), and health (health situation and abilities to deal with daily life concerns). All items were scored on a seven-point scale from dissatisfaction to satisfaction (0—*very dissatisfied*; 1—*dissatisfied*; 2—*mostly dissatisfied*; 3—*mixed (about equally satisfied and dissatisfied)*; 4—*mostly satisfied*; 5—*satisfied*; 6—*very satisfied*). The BMLSS score was referred to as a 100% level (transformed scale score). The internal consistency of the instrument was found to be good in the validation study (Cronbach's alpha = 0.87). In this sample, Cronbach's alpha = 0.82.

#### Perception of Burden

Perceived restrictions of daily life, of being under pressure/stressed, anxiety/insecurity, loneliness/social isolation, and financial-economic situation due to corona pandemic were measured with five numeric rating scales (5NRS), ranging from 0 (*not at all*) to 100 (*very strong*) as described (8). These five variables can be combined to a factor termed “Perceived burden” (“Stressors”) with good internal consistency (Cronbach's alpha = 0.80).

#### Indicators of Spirituality

To measure reliance on faith, item A37 from the Reliance on God's Help scale (38) was used as a differentiating variable to assess intrinsic religiosity in terms of an attitude. It states “faith as a stronghold in difficult times” and can be scored on a three-point scale (0—*disagreement*; 2—*indifference*; 3—*agreement*). The frequency of spiritual/religious practices such as meditation or praying was assessed with a four-grade scale ranging from *never*, to *at least once per month, at least once per week*, and *at least once per day* as described (2, 8).

#### Physical Activities

We addressed physical activity/sporting and walking outside in nature with a four-grade scale (*never, at least once per month, at least once per week*, and *at least once per day*) as described (2, 8). When awe is indeed triggered by experience in nature, etc., then a positive association with walking outside in nature can be expected, but intuitively, not that intensely, with physical activity/sporting.

### Statistical Analyses

Descriptive statistics for demographic variables and for factors are presented as frequencies for categorical variables and mean (±standard deviation, SD) for numerical variables. Analyses of variance (ANOVA) as well as first-order correlation (Spearman rho) and linear regression analyses with stepwise variable selection method based on probabilities (*p*-values) were computed with SPSS 23.0. To investigate possible interactions as mediation and moderation of independent variables on dependent factors, the Mediation and Moderation Analyses were performed with software R (4.0.3) packages “mediation” (39) and “olsrr” (40). Given the exploratory character of this study, we set a stricter significance level at *p* < 0.01 (41). With respect to classifying the strength of the observed correlations, we adjusted the recommended thresholds (42) to *r* > 0.5 as a strong correlation, an *r* between 0.3 and 0.5 as a moderate correlation, an *r* between 0.2 and 0.3 as a weak correlation, and *r* < 0.2 as negligible or no correlation. Cohen's d effect sizes were used to report differences between groups (43).

## Results

### Description of the Sample

Within the sample (*N* = 2.573), women (68%) and persons with a Christian affiliation (74%) were predominating (Table 1). Participants' mean age was 48.7 ± 14.2 years. Their area of profession was heterogeneous, ranging from Administration, Economy, Education, Medicine/Health, Church, and other (incl. students and retired persons). Despite a predominance of Christian and other religious affiliations (78%), only 43% stated to have faith as a stronghold in difficult times, 29% were undecided, and 28% disagreed. In line with this proportion, 38% were praying at a daily level (indicating a religious person) and 22% meditating at a daily level, while 44% were never meditating and 35% never praying. Physical activities/sporting and walking outside in the nature were practiced by 71 and 70%, respectively, at least once per week (Table 1).

**Table 1:** Sociodemographic data of participants (*N* = 2,573).

	***n***	**% of responders**	**Mean ± SD**	**Range**
**Gender**				
Women	1,443	67.9		
Wen	821	32		
**Age (years)**	1,261		48.7 ± 14.2	15–92
**Living conditions**				
Family household	1,664	64.7		
Shared house	229	8.9		
Single	515	20		
Monastery/community	180	7		
Profession*				
Administration	368	14.3		
Economy	243	9.4		
Education	285	11.1		
Medicine/health	527	20.5		
Church	434	16.9		
Other	913	35.6		
**Religious affiliation**				
Catholics	1,331	51.7		
Protestant	569	22.6		
Other	105	4.1		
None	570	22.2		
**Faith as stronghold in difficult times**				
Disagreement	705	28,1		
Undecided	728	29.1		
Agreement	1,072	42.8		
**Meditation**			1.2 ± 1.2	0–3
Never	1,054	44.3		
At least once per month	345	14.5		
At least once per week	449	18.9		
At least once per day	530	22.3		
**Praying**			1.6 ± 1.3	0–3
Never	821	34.6		
At least once per month	267	11.3		
At least once per week	378	15.9		
At least once per day	905	38.2		
**Physical activity/sporting**			1.8 ± 0.9	0–3
Never	354	14.9		
At least once per month	348	14.6		
At least once per week	1,217	51.1		
At least once per day	461	19.4		
**Walking outside in nature**			2.1 ± 0.8	0–3
Never	83	3.5		
At least once per month	388	16.2		
At least once per week	1,236	51.6		
At least once per day	690	18.8		
**Wellbeing and burden**				
Life satisfaction (BMLSS-10)	2,573		67.2 ± 16.1	0–100
Satisfaction with Support (BMLSS Support)	2,571		60.6 ± 18.2	0–100
Wellbeing (WHO-5 100)	2,572		58.7 ± 22.2	0–100
Wellbeing (WHO-5 sum)	2,573		14.7 ± 5.5	0–25
Low wellbeing (WHO-5 sum scores <13)	835	32.5		
Moderate wellbeing (WHO-5 sum scores 13–18)	973	37.8		
High wellbeing (WHO-5 sum scores >18)	765	29.7		
Perceived burden (“Stressors”) (5NRS)	2,572		31.0 ± 20.9	0–100
Loneliness/social isolation (NRS)	2,572		25.0 ± 28.5	0–100
No loneliness (NRS scores = 0)	900	35		
Low to moderate loneliness (NRS cores 10–50)	1,245	48.4		
High loneliness (NRS scores 50–100)	427	16.6		
**Perceived changes (PCQ)**				
Nature/Silence/Contemplation	2,549		56.6 ± 21.0	0–100
Spirituality	2,549		41.8 ± 26.0	0–100
Relationships	2,551		63.3 ± 18.9	0–100
Reflection on life	2,549		52.3 ± 25.0	0–100
Digital media usage	2,548		54.5 ± 23.7	0–100

* *Some ascribed themselves to multiple professions, and thus, the response rate is >100%*.

Within the sample, life satisfaction and well-being were in a moderate range. Thirty-two percent have WHO-5 scores <13, indicating low well-being. Furthermore, 17% have strong feelings of loneliness/social isolation. However, participants' general perceived burden (5NRS) scored in the lower third, indicating that the majority of the participants were (only) “somewhat” affected (Table 1).

Participants perceived changes in their attitudes and behaviors due to the Corona pandemic, particularly with respect to *Nature/Silence/Contemplation* and *Relationships*, while changes in *Digital media usage* and *Reflection of life* were less strong; in contrast, changes in *Spirituality* were rather not perceived (Table 1).

### Perception of Awe and Gratitude

Within the sample, stopping and being “captivated by the beauty of nature” and “experience and value of beauty” were experienced most often (often to very often by 90 and 86%, respectively), followed by feelings of “great gratitude” (often to very often by 77%), while feelings of “wondering awe” were experienced less often (often to very often by 47%) (Table 2).

**Table 2:** Items and response rate of the Awe/Gratitude scale.

		**Never (%)**	**Seldom (%)**	**Often (%)**	**Very often (%)**	**Mean score (0–3)**
ED1	I have a feeling of great gratitude.	3	21	52	25	1.99 ± 0.75
ED2	I have a feeling of wondering awe.	10	44	36	11	1.48 ± 0.82
ED3	I still have learned to experience and value beauty.	1	9	57	33	2.23 ± 0.63
ED4	I stop and am captivated by the beauty of nature.	1	13	46	40	2.24 ± 0.71
ED5	I pause and stay spellbound at the moment.	3	38	43	17	1.74 ± 0.76
ED6	In certain places I become very quiet and devout.	3	31	46	21	1.84 ± 0.78
ED7	I stop and then think of so many things for which I am really grateful.	3	28	48	21	1.88 ± 0.77

As shown in Table 3, Awe/Gratitude scored significantly higher particularly among women (Cohen's *d* = 0.40), older persons (*d* = 0.88), persons who rely on their faith as a “stronghold in difficult times” (*d* = 0.99), religious brothers and sisters (*d* = 0.60), those with higher well-being (*d* = 0.70), and lower perceptions of loneliness (*d* = 0.49). Further, Awe/Gratitude was moderately related to a person's meditation and praying frequency, and weakly to life satisfaction (particularly satisfaction with life in general, *r* = 0.30) and to walking outside in nature, and marginally only to physical activities/sporting (Table 4).

**Table 3:** Awe/Gratitude in different subgroups.

	**Awe/Gratitude**
All	63.74 ± 18.79
**Gender**	
Female	66.13 ± 18.32
Male	58.72 ± 18.79
*F* value	89.85
*p* value	<0.0001
Cohen's d (f/m)	0.40
**Age cohorts**	
<30 years	57.10 ± 17.92
30–40 years	57.20 ± 19.27
41–50 years	62.25 ± 19.06
51–60 years	67.04 ± 17.84
61–70 years	69.03 ± 16.96
>70 years	72.52 ± 16.62
*F* value	37.90
*p* value	<0.0001
Cohens' d (<30/>70)	0.88
**Religious**	
Living in Monastery	73.02 ± 15.32
All other	63.04 ± 18.84
*F* value	48.11
*p* value	<0.0001
Cohens' d (monastery/not)	0.60
**Faith as stronghold**	
Does not apply	53.67 ± 18.11
Partly	62.00 ± 16.25
Applies	71.36 ± 17.58
*F* value	225.56
*p* value	<0.0001
Cohens' d (yes/no)	0.99
**Well-being (WHO-5)**	
Scores <13	57.03 ± 18.50
Scores 13–18	63.85 ± 16.94
Scores >18	70.92 ± 18.68
*F* value	119.05
*p* value	<0.0001
Cohens' d (high/low)	0.70
**Loneliness (NRS)**	
Scores 50–100	57.67 ± 19.95
Scores 10–50	63.23 ± 17.17
Scores = 0	67.32 ± 19.56
*F* value	40.31
*p* value	<0.0001
Cohens' d (high/low)	0.49

**Table 4:** Correlations between Awe/Gratitude and other variables.

	**Awe/Gratitude**
**Perceived Changes (PCQ)**	
Nature/Silence/Contemplation	0.408**
Spirituality	0.407**
Relationships	0.333**
Reflection on life	0.257**
Digital media usage	0.146**
Restrictions	−0.174**
**Well-being and burden**	
Life satisfaction (BMLSS-10)	0.289**
Well-being (WHO-5)	0.316**
Perceived burden (5NRS)	−0.153**
**Frequency of activities**	
Physical activities/sporting	0.143**
Walking outside in nature	0.237**
Meditation	0.442**
Praying	0.365**

** *p < 0.001 (Spearman rho); moderate associations are highlighted*.

### Relation of Awe/Gratitude to Perceived Changes Due to the Corona Pandemic

With respect to perceived changes in attitudes and behaviors because of the Corona pandemic, Awe/Gratitude was moderately related to *Nature/Silence/Contemplation, Spirituality* and *Relationships*, weakly also to *Reflection of life*, and marginally only *to Digital media usage* or perceived *Restrictions* (Table 4). Also, Perceived burden (“Stressors”) related to the Corona pandemic was marginally (and inversely) related to Awe/Gratitude scores.

### Predictors of Awe/Gratitude

As there are several variables significantly related to perceptions of Awe/Gratitude, both inherent and in response to the pandemic, regression analyses were performed to analyze which independent variables would predict Awe/Gratitude (as a dependent variable). These regression analyses were performed in different steps, which refer to previous findings (8): (1) including gender and age as independent variables, (2) adding spirituality-related variables (faith as a strong hold, meditation, and praying), (3) adding well-being and life satisfaction (which is related also to the feelings during the pandemic), and (4) adding perceived changes and burden because of the pandemic.

As shown in Table 5, higher age and female gender predicted 11% of Awe/Gratitude score variance (with age being the best predictor). Adding the three spirituality-related variables, the explained variance increased to 28% (now meditation frequency became the best predictor). Adding well-being and life satisfaction to the model in the third step, the included variables would explain 35% of variance (now age loses its relevance as a predictor). Adding the topics of perceived changes and perceived burden due to the corona pandemic raises the predictive power to 40%. Finally, the best predictors of Awe/Gratitude were frequency of meditation practice, female gender, life satisfaction and well-being, faith as a stronghold, perceived burden, and life reflection because of the pandemic. Perceived *Restrictions* and changes in spiritual practices and perceptions (*Spirituality*), and changed *Digital media usage* had no independent predictive relevance in this final model.

**Table 5:** Predictors of well-being as dependent variable (regression analyses).

	**Beta**	**T**	***p***	**Beta**	**T**	***p***	**Beta**	**T**	***p***	**Beta**	**T**	***p***
**Dependent variable: GrAw-7 scores**	**Model 1:** ***F*** **=** **135.6**. ***R***^****2****^ **=** **0.11**			**Model 2:** ***F*** **=** **157.7**. ***R***^****2****^ **=** **0.28**			**Model 3:** ***F*** **=** **154.6**. ***R***^****2****^ **=** **0.35**			**Model 4:** ***F*** **=** **95.5** ***R***^****2****^ **=** **0.40**		
Constant		32.891	<0.0001		33.405	<0.0001		19.082	<0.0001		8.355	<0.0001
Male gender	−0.199	−10.081	<0.0001	−0.177	−9.300	<0.0001	−0.190	−10.482	<0.0001	−0.162	−9.157	<0.0001
Age cohorts	0.266	13.450	<0.0001	0.100	4.931	<0.0001	0.070	3.560	<0.0001	0.054	2.842	0.005
Faith as hold				0.180	6.669	<0.0001	0.144	5.584	<0.0001	0.105	3.972	<0.0001
Meditation				0.286	13.393	<0.0001	0.285	13.963	<0.0001	0.232	11.180	<0.0001
Praying				0.080	2.911	0.004	0.088	3.333	0.001	0.075	2.763	0.006
Well-being (WHO-5)							0.134	5.945	<0.0001	0.161	6.721	<0.0001
Life satisfaction (BMLSS-10)							0.164	7.382	<0.0001	0.184	8.019	<0.0001
Nature/Silence/Contemplation (PCQ)										0.084	3.190	0.001
Spirituality (PCQ)										0.025	0.805	0.421
Relationships (PCQ)										0.082	3.668	<0.0001
Reflection on life (PCQ)										0.101	4.232	<0.0001
Digital media usage (PCQ)										−0.020	−1.048	0.295
Perceived restrictions (PCQ)										−0.035	−1.582	0.114
Perceived burden (5NRS)										0.103	4.210	<0.0001

### Predictors of Well-Being

Which of the analyzed variables would predict best a persons' well-being (as dependent variable) during the Corona pandemic? Awe/Gratitude (as an influencing variable) alone would predict 9% of participants' variance in their well-being, as regression analyses indicated. Stepwise regression analyses including the abovementioned independent variables revealed that it is foremost life satisfaction and low perceived burden (related to the pandemic), which would together explain 44% of well-being variance. Next, relevant variables were Awe/Gratitude and *Nature/Silence/Contemplation* (PCQ), which would add 3% of the explained variance. The next six significant predictors (low *Reflection of life*, higher age, walking outside in nature, low *Relationships*, female gender, physical activities/sporting) were of even less relevance as all of them together would add only 2% of the explained variance (Table 6). Not significant in this prediction model were frequency of Meditation and Praying, *Spirituality* (PCQ), and *Digital media usage* (PCQ).

**Table 6:** Predictors of well-being (stepwise regression).

**Dependent Variable:**			
**Well-being (WHO-5)**			
**Model 10: *F* = 196.3, *p* < 0.0001; *R*^**2**^ = 0.49**	**Beta**	**T**	***p***
(constant)		5.984	<0.0001
Life satisfaction (BMLSS-10)	0.339	17.154	<0.0001
Perceived burden (5NRS)	−0.320	−16.578	<0.0001
Awe/Gratitude (GrAw-7)	0.123	6.535	<0.0001
Perceived Changes: Nature/Silence/Contemplation (PCQ)	0.168	7.251	<0.0001
Perceived Changes: Reflection of life (PCQ)	−0.115	−5.651	<0.0001
Age cohort	0.062	3.715	<0.0001
Walking outside in nature	0.049	2.907	0.004
Perceived Changes: Relationships (PCQ)	−0.059	−2.912	0.004
Female gender	0.046	2.801	0.005
Physical activities/sporting	0.039	2.407	0.016

*Not significant in the model: Praying, Meditation, Satisfaction with support, Perceived Changes: Spirituality; Perceived Changes: Digital media usage*.

### Meditation and Moderator Analyses

**Figure 1:**
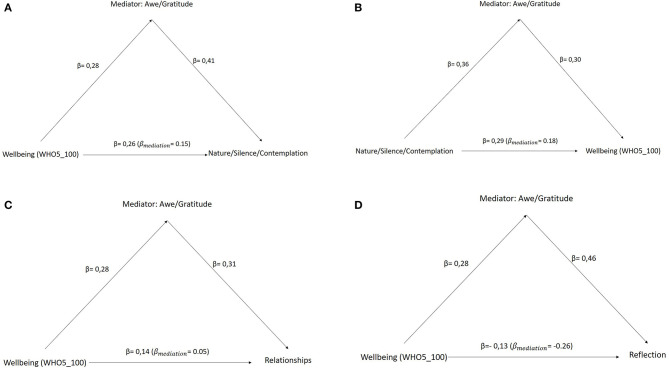
Different mediation models with Awe/Gratitude as mediator. Well-being as predictor and Nature/Silence/Contemplation as outcome mediated by Awe/Gratitude **(A)**. The proportion of causal effect explained by the mediator is 42%, direct effect is 0.15, and total effect is 0.26. Nature/Silence/Contemplation as predictor and well-being as outcome mediated by Awe/Gratitude **(B)**. The proportion of causal effect explained by the mediator is 38%, direct effect is 0.18, and total effect is 0.29. Well-being as predictor and Relationships as outcome mediated by Awe/Gratitude **(C)**. The proportion of causal effect explained by the mediator is 62%, direct effect is 0.05, and total effect is 0.14. Well-being as predictor and Reflection as outcome mediated by Awe/Gratitude **(D)**. The proportion of causal effect explained by the mediator is 95%, direct effect is −0.26, and total effect is −0.13.

It is supposed that Awe/Gratitude could play a role in the analysis either as mediator or moderator. For this purpose, both causal relationships will be investigated. Mediation analysis describes a causal sequence of effects from the predictor variable on the outcome. Moderator analysis evaluates if a given variable affects the direction and/or strength of the causal relationship (in terms of an “enhancer” or “buffer”). Mediation analysis revealed that Awe/Gratitude mediates 42% (*p* < 0.001) of the link between well-being as a predictor on *Nature/Silence/Contemplation* as an outcome and has a direct effect of β = 0.15 (*p* < 0.001) and an indirect effect of β = 0.11 (*p* < 0.001) (Figure 1A). Furthermore, Awe/Gratitude mediates 38% (*p* < 0.001) of the link between *Nature/Silence/Contemplation* as a predictor on well-being as the outcome; the direct effect is β = 0.18 (*p* < 0.001), and the indirect effect is β = 0.11 (*p* < 0.001) (Figure 1B). For *Relationships* as an outcome, Awe/Gratitude mediates 62% (*p* < 0.001) of the effect of well-being as predictor, with small direct and indirect effects of β = 0.05 (*p* < 0.01) and β = 0.08 (*p* < 0.001), respectively (Figure 1C). Finally, Awe/Gratitude mediates 95% (*p* < 0.001) of the effect of well-being on *Reflection of life*; interestingly, the direct effect β = −0.26 (*p* < 0.001) has a negative influence decreasing the intensity of *Reflections*, while the indirect effect β = 0.12 (*p* < 0.001) remains positive (Figure 1D). All other perceived changes were not significantly mediated by Awe/Gratitude. However, Awe/Gratitude was a significant mediator, but not a significant moderator of the link between perceived changes and well-being (data not shown).

**Figure 2:**
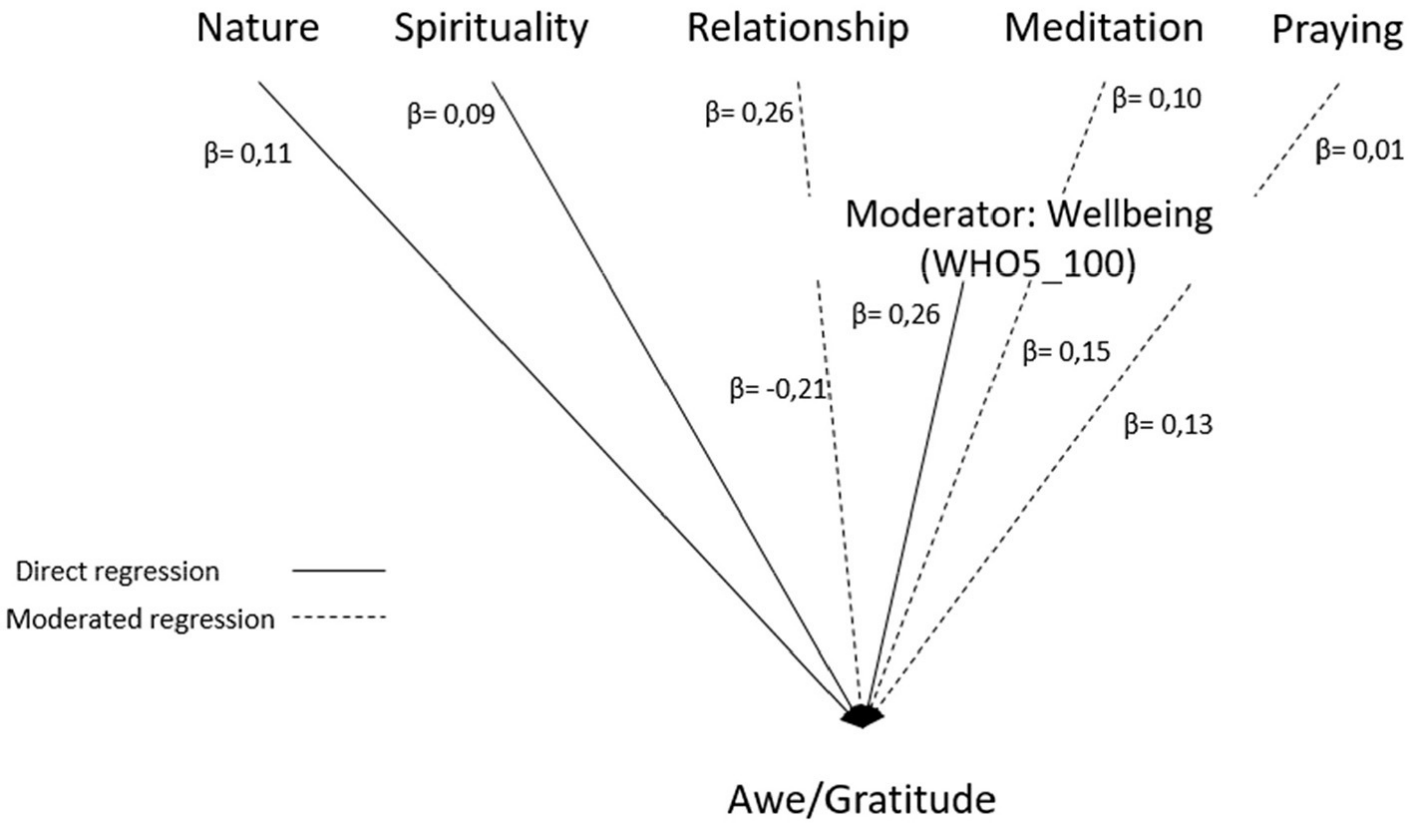
Moderator model for Awe/Gratitude (GrAw), *R*^2^ = 0.36, with well-being (WHO-5) negatively moderating the regression effect of Relationship, but positively moderating the effect of Meditation and Praying on Awe/Gratitude.

Next, we analyzed whether well-being could moderate interaction effects between perceived changes (namely, *Nature/Silence/Contemplation, Spirituality*, and *Relationships*, and also Meditation or Praying) on Awe/Gratitude. Interestingly, Awe/Gratitude can be estimated through the variables *Nature/Silence/Contemplation* (β = 0.11), *Spirituality* (β = 0.09), *Relationships* (β = 0.26), well-being (β = 0.26), and meditation frequency (β = 0.10), but not significantly through frequency of praying (β = 0.01). The β values represent the standardized estimates of the regression model and are in the interval (0,1). In this model, well-being was moderating weakly the relationship between Awe/Gratitude and meditation (β = 0.15), praying (β = 0.13) and inversely also *Relationship* (β = −0.21), but not *Spirituality* or *Nature/Silence/Contemplation* (Figure 2). The respective model has a moderate adequacy explaining 36% of the variance in the data. Furthermore, no significant moderation model was observed for well-being as response variable (data not shown).

### Course of Well-Being, Perceived Burden, and Awe/Gratitude Within the 6-Month Observation Period

**Figure 3:**
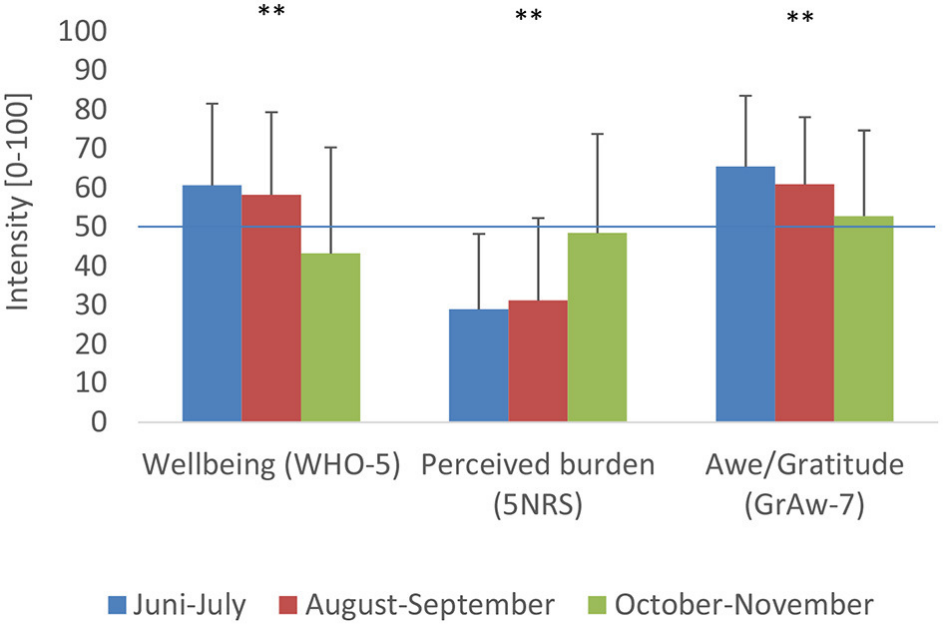
Course of well-being, perceived burden, and awe/gratitude within the 6-month observation period (**p < 0.0001; Spearman rho).

Time of course may have significant influence on several variables. The main recruiting phase was within June and July 2020 (*n* = 2,046); nevertheless, we further recruited participants within the summer months August and September (*n* = 288) and during the autumn months October and November (*n* = 242), which went along with the start of the second wave of the COVID-19 pandemic in Germany. Referring to different cohorts, within these 6 months, participants' well-being decreased significantly [*F*_(2, 2, 575)_ = 70.4, *p* < 0.0001], while Perceived burden increased [*F*_(2, 2, 574)_ = 100.8, *p* < 0.0001] (Figure 3). Within this time span, feelings of Awe/Gratitude were significantly declining [*F*_(2, 2, 572)_ = 54.6, *p* < 0.0001]. This decline is more related to the decrease in well-being rather than to the increase in Perceived burden, as well-being and Awe/Gratitude are moderately correlated (*r* = 0.32, *p* < 0.001; Spearman rho), while the association between Perceived Burden and Awe/Gratitude is marginal only (*r* = –0.15, *p* < 0.001; Spearman rho). It has to be noted, however, that the participants of October to November are, on average, 5 years younger (mean age) than the participants of the months June to July [*F*_(2, 2, 546)_ = 28.6, *p* < 0.0001], while the gender proportion was similar (*p* = 0.68, Chi^2^).

## Discussion

### Answers to Our Research Questions

Referring to our research questions, first, we can state that particularly women, older persons, and religious/spiritual persons perceived Awe/Gratitude more often (and intensely); this is also true for those with higher well-being and lower perceptions of loneliness. It seems that both, low scores of well-being and feelings of loneliness may distract a person's awareness for the Sacred and the “beauty” in life. In persons with depressive states, their perception of beauty (in nature) was found to be lower compared with those of the other patients, while their general ability to stand in wondering awe and to be grateful was not significantly different (19). In this study, well-being was particularly related to feelings of gratefulness (item ED1: *r* = 0.33) and to the experience and value of beauty (ED3: *r* = 0.30), to stopping in awe and then thinking “of so many things for which I am really grateful” (ED7: *r* = 0.28). Thus, low emotional well-being is associated with low feelings (or felt reasons) of gratefulness in life, in general—and probably also during the pandemic, in particular. In this study, reduced well-being was strongly associated with dissatisfaction with oneself (*r* = 0.56), dissatisfaction with life, in general (*r* = 0.54), and dissatisfaction with own abilities to cope with daily life situations (*r* = 0.50). These more “personal” variables could be related to the underlying personality structure.

With respect to perceived changes during the pandemic as second focus, more intense feelings of Awe/Gratitude were particularly related to *Nature/Silence/Contemplation, Spirituality* and *Relationships*. This means that the general ability to experience Awe/Gratitude particularly during the Corona pandemic may sensitize to perceive the world around (including nature and concrete persons) more intensely. Furthermore, well-being modified the relation between *Relationships* and Awe/Gratitude decreasing the intensity of those feelings. This interaction indicates that *Relationships* might be a protective factor, but if well-being fluctuates in a lower range of scores, the potency of Awe/Gratitude degenerates. Apart from moderation effects, we found evidence that Awe/Gratitude is mediating the link between *Nature/Silence/Contemplation* and well-being and also in the reverse order between well-being and *Nature/Silence/Contemplation*. This means that the effect of well-being on the perception of changes related to nature, silence, and contemplation can be enhanced by Awe/Gratitude, on the one hand, and that the perceived changes related to nature, silence, and contemplation may positively influence a person's well-being, mediated by the ability to perceived Awe/Gratitude. Moreover, Awe/Gratitude weakly mediates the relationship between well-being and *Relationships* and negatively between well-being and *Reflection* of life. Well-being itself is a buffer for *Reflection of life* (which implies worrying thoughts about meaning and purpose in life and the lifetime one has, and more intensive perceptions of loneliness) and the mediator and may thus reduce the intensity of this effect, acting as an enhancer.

The underlying dynamics for the realization of this ability to perceive beauty in nature, to stand in wondering awe and finally to be grateful might be comparable or even the same as in posttraumatic growth (27, 28) or spiritual transformation (29, 30), processes in which people change their attitudes and make new resolutions because of specific experiences. Yaden et al. (15) likewise observed that the experience of awe may result in feelings of connectedness with others and nature. The realization of such dynamics seems to depend on various factors. The best predictors of Awe/Gratitude were the frequency of meditation (which may indicate that the awareness can be trained), female gender (women are usually more aware of their emotions and more sensitive toward spiritual issues) (44), life satisfaction, and well-being (which may indicate that positive emotional states may facilitate awareness), faith as a stronghold (which may imply that whatever may come, one has unconditional trust in God or another source of hope), and inversely and marginally only perceived burden and also life reflection (which has a negative worrying connotation in this context), too. *Nature/Silence Contemplation* and *Relationships* had a further, but weaker, impact on Awe/Gratitude as a dependent variable.

The third research question was whether or not feelings of awe and subsequent gratitude contribute to participants' well-being, as awe was suggested to increase well-being and personal change (45) and to be related to openness and extroversion as a personality structure (46). In our study, Awe/Gratitude was indeed moderately associated with well-being and would predict 9% of participants' well-being variance. Best predictors of participants' emotional well-being were multidimensional life satisfaction and low perceived burden (related to the pandemic), and further Awe/Gratitude and *Nature/Silence/Contemplation*. Thus, these perceptions have their role in relation to well-being, but not in the forefront.

Even when it is true that Awe/Gratitude facilitates to be more aware of positive changes in attitudes and behaviors due to the COVID-19 pandemic, it, nevertheless, does not relevantly buffer against the perceived burdens and restrictions as the associations are marginal only. Moderator analyses further indicated that Awe/Gratitude was not a significant moderator of the link between perceived changes and well-being. It cannot be called a buffering resilience factor, but rather an ability to perceive the positive aspects in life—in spite of the stressors. Actually, this could be considered an interesting process of inner development similar or closely related to the concept of mindfulness: being aware of the situation as it is, and deal with the situation as it is without judgment, as judgmental processes would result in negative emotions (47–49).

The results show that the practice of meditation and praying is related to Awe/Gratitude and (in terms of training) may sensitize to be more aware of the underlying moments and situations that cause feelings of wondering awe, and thus, it was of interest whether well-being could moderate these pathways. We found that the effect of both spiritual practices (meditation and praying), when moderated by a person's well-being, increases the levels of Awe/Gratitude more than if they were evaluated separately, while the interaction between more intense *Relationship* and well-being decreases Awe/Gratitude scores. This result could be due to a range of participants who reported lower well-being but high scores for perceived changes in terms of more intense *Relationship* (which may become more relevant as a stabilizing resource). The variables with the strongest contribution to higher levels of Awe/Gratitude were perceived changes in *Relationships* and well-being, and the strongest moderation was observed between frequency of meditation practices and well-being. Praying as a separate variable did not contribute to the model.

### Limitations

We are aware that the data are not representative for all parts of German society, as the recruitment process may have favored persons with Internet access, academic contexts, and persons with a Christian background. However, while this selection bias is acceptable to address the research questions, it would be interesting to analyze persons from others contexts.

Due to the cross-sectional design of the study, no causal conclusions can be drawn. To account for this, we added data from different recruitment months (resulting in different cohorts). Data from these cohorts (Figure 3) indicate that a decrease in well-being and Awe/Gratitude may precede an increase in perceived burden. However, one cannot fully exclude the possibility that perceptions of Awe are better triggered during spring times instead of late autumn.

## Conclusions

Perceptions of Awe and subsequent Gratitude are higher in persons with a religious background (11), and in those with more intense meditation or prayer practice. Such spiritual practices may facilitate these perceptions in terms of “training” and attitude. However, these experiences of Awe and Gratitude do not usually buffer against adverse events in life and cannot prevent perceived burden due to the pandemic; rather, they facilitate to, nevertheless, perceive the positive aspects of life, particularly *Nature/Silence/Contemplation, Spirituality*, and *Relationships*. This indicates higher awareness of a connectedness with the world around and with concrete others (horizontal direction of relations) and with the Sacred (vertical direction of relations). As Awe/Gratitude is further mediating the effects of *Nature/Silence/Contemplation* on well-being, intervention programs to train these perceptions could be considered in order to support people particularly in the time of the COVID-19 pandemic, as these self-transcendent feelings are also related to prosocial behaviors with respectful treatment of others and commitment to persons in need (17).

## Data Availability Statement

According to the data protection regulations, the data set cannot be made publicly available. Data are however available from the first author upon reasonable request.

## Ethics Statement

Ethical review and approval was not required for the study on healthy human participants in accordance with the local legislation and institutional requirements. Written informed consent for participation was not required for this study in accordance with the national legislation and the institutional requirements. Participants were assured confidentially and were informed about the purpose of the study and data protection information at the starting page of the online survey. By filling in the anonymous questionnaire, interested persons consented to participate.

## Author Contributions

AB designed the study, set up the online survey, and wrote the first draft of the paper. DR and AB undertook the statistical analyses. KB and JS were actively involved in writing and revising the manuscript. All authors provided feedback and approved the final manuscript.

## Conflict of Interest

The authors declare that the research was conducted in the absence of any commercial or financial relationships that could be construed as a potential conflict of interest.

## References

[1] Büntzel J Klein M Keinki C Walter S Büntzel J Hübner J. Oncology services in corona times: a flash interview among German cancer patients and their physicians. J Cancer Res Clin Oncol. (2020) 146:2713–5. 10.1007/s00432-020-03249-z 32415341 PMC7226710

[2] Büssing A Hübner J Walter S Gießler W Büntzel J. Tumor patients' perceived changes of specific attitudes, perceptions and behaviors due to the Corona pandemic and its relation to reduced wellbeing. Front Psychiatry. (2020) 11:574314. 10.3389/fpsyt.2020.574314 33192703 PMC7581913

[3] Campos-Mercade P Meier MN Schneider FH Wengström E. Prosociality Predicts Health Behaviorsduring the COVID-19 Pandemic. ECON - Working Papers 346. Department of Economics - University of Zurich. Available online at: https://ideas.repec.org/p/zur/econwp/346.html (accessed February 22, 2021).

[4] Scheid JL Lupien SP Ford GS West SL. Commentary: physiological and psychological impact of face mask usage during the COVID-19 pandemic. Int J Environ Res Public Health. (2020) 17:6655. 10.3390/ijerph17186655 32932652 PMC7558090

[5] Mukhtar S. Psychological health during the coronavirus disease 2019 pandemic outbreak. Int J Soc Psychiatry. (2020) 66:512–6. 10.1177/0020764020925835 32434402 PMC7405632

[6] Salari N Hosseinian-Far A Jalali R Vaisi-Raygani A Rasoulpoor S Mohammadi M. Prevalence of stress, anxiety, depression among the general population during the COVID-19 pandemic: a systematic review and meta-analysis. Global Health. (2020) 16:57. 10.1186/s12992-020-00589-w 32631403 PMC7338126

[7] Shader RI. COVID-19 and depression. Clin Ther. (2020) 42:962–3. 10.1016/j.clinthera.2020.04.010 32362345 PMC7184005

[8] Büssing A Recchia DR Hein R Dienberg T. Perceived changes of specific attitudes, perceptions and behaviors during the Corona pandemic and their relation to wellbeing. Health Qual Life Outcomes. (2020) 18:374. 10.1186/s12955-020-01623-6 33256755 PMC7702679

[9] James W. The Varieties of Religious Experience. A Study of Human Nature. Introduction by Reinhold Niebuhr. New York: Touchstone (1997).

[10] Büssing A Rechia DR Baumann K. Validation of the gratitude/awe questionnaire and its association with disposition of gratefulness. Religions. (2018) 9:117. 10.3390/rel9040117

[11] Büssing A. Ehrfurcht/Dankbarkeit als säkulare Form der Spiritualität bei jungen Erwachsenen und Ordens-Christen. Spiritual Care. (2020) 9:3–11. 10.1515/spircare-2019-0057

[12] Keltner D Haidt J. Approaching awe, a moral, spiritual, and aesthetic emotion. Cogn Emotion. (2003) 17:297–314. 10.1080/02699930302297 29715721

[13] Pearsall P. Awe: The Delights and Dangers of Our Eleventh Emotion. Deerfield Beach, FL: Health Communications, Inc. (2007).

[14] Silvia PJ Fayn K Nusbaum EC Beaty RE. Openness to experience and awe in response to nature and music: personality and profound aesthetic experiences. Psychol Aesthetic Creat Arts. (2015) 9:376–84. 10.1037/aca0000028

[15] Yaden DB Kaufman SB Hyde E Chirico A Gaggioli A Zhang JW. The development of the Awe Experience Scale (AWE-S): a multifactorial measure for a complex emotion. J Posit Psychol. (2018) 14:474–88. 10.1080/17439760.2018.1484940

[16] Prade C Saroglou V. Awe's effects on generosity and helping. J Posit Psychol. (2016) 11:1–9. 10.1080/17439760.2015.112799226640507

[17] Büssing A Recchia DR Dienberg T. Attitudes and behaviors related to Franciscan-inspired Spirituality and their associations with compassion and altruism in Franciscan brothers and sisters. Religions. (2018) 9:324. 10.3390/rel9100324

[18] Van Cappellen P Saroglou V. Awe activates religious and spiritual feelings and behavioral intentions. Psychol Relig Spirituality. (2012) 4:223–36. 10.1037/a0025986

[19] Büssing A Wirth AG Reiser F Zahn A Humbroich K Gerbershagen K. Experience of gratitude, awe and beauty in life among patients with multiple sclerosis and psychiatric disorders. Health Qual Life Outcomes. (2014) 12:63. 10.1186/1477-7525-12-63 24779860 PMC4029818

[20] Krause N Hayward RD. Assessing whether practical wisdom and awe of God are associated with life satisfaction. Psychol Relig Spirituality. (2015) 7:51–9. 10.1037/a0037694

[21] Wood AM Froh JJ Geraghty AW. Gratitude and well-being: a review and theoretical integration. Clin Psychol Rev. (2010) 30:890–905. 10.1016/j.cpr.2010.03.005 20451313

[22] Kendler KS Liu XQ Gardner CO McCullough ME Larson D Prescott CA. Dimensions of religiosity and their relationship to lifetime psychiatric and substance use disorders. Am J Psych. (2003) 160:496–503. 10.1176/appi.ajp.160.3.496 12611831

[23] Lambert NM Fincham FD Stillman TF. Gratitude and depressive symptoms: the role of positive reframing and positive emotion. Cogn Emot. (2012) 26:615–33. 10.1080/02699931.2011.595393 21923564

[24] Watkins PC Xiong I Kolts RI. How grateful processing brings closure to troubling memories. Paper Presented at the 20th Annual Convention of the Association for Psychological Science. Chicago, I. L. (2008).

[25] Diessner R Lewis G. Further validation of the Gratitude, Resentment, and Appreciation Test (GRAT). J Soc Psychol. (2007) 147:445–7. 10.3200/SOCP.147.4.445-448 17955754

[26] Li L Li S Wang Y Yi J Yang Y He J. Coping profiles differentiate psychological adjustment in Chinese women newly diagnosed with breast cancer. Integr Cancer Ther. (2017) 16:196–204. 10.1177/1534735416646854 27154183 PMC5739123

[27] Ai AL Hall D Pargament K Tice TN. Posttraumatic growth in patients who survived cardiac surgery: the predictive and mediating roles of faith-based factors. J Behav Med. (2013) 36:186–98. 10.1007/s10865-012-9412-6 22460360

[28] Tedeschi RG Shakespeare-Finch J Taku K Calhoun LG. Posttraumatic growth. Theory, research, and applications. Milton Park: Taylor and Francis (2018). 10.4324/9781315527451

[29] Kremer H Ironson G. Everything changed: spiritual transformation in people with HIV. Int J Psychiatry Med. (2009) 39:243–62. 10.2190/PM.39.3.c 19967898

[30] Ironson G Kremer H. Spiritual transformation, psychological well-being, health, and survival in people with HIV. Int J Psychiatry Med. (2009) 39:263–81. 10.2190/PM.39.3.d 19967899

[31] Atamba C. Restorative effects of awe on negative affect after receiving negative performance feedback. J Psychol Afr. (2019) 29:95–103. 10.1080/14330237.2019.1594640

[32] Koh AHQ Tong EMW Yuen AYL. The buffering effect of awe on negative affect towards lost possessions. J Posit Psychol. (2017) 14:1–10. 10.1080/17439760.2017.1388431

[33] Rankin K Andrews SE Sweeny K. Awe-full uncertainty: easing discomfort during waiting periods. J Posit Psychol. (2019) 15:1–10. 10.1080/17439760.2019.1615106

[34] Algoe SB Stanton AL. Gratitude when it is needed most: social functions of gratitude in women with metastatic breast cancer. Emotion. (2012) 12:163–8. 10.1037/a0024024 21707160

[35] Seligman MEP. Authentic Happiness. Using the Positive Psychology to Realize Your Potential for Lasting Fulfillment. New York, NY: The Free Press (2002).

[36] Bech P Olsen LR Kjoller M Rasmussen NK. Measuring well-being rather than the absence of distress symptoms: a comparison of the SF-36 mental health subscale and the WHO-Five well-being scale. Int J Methods Psychiatr Res. (2013) 12:85–91. 10.1002/mpr.145 12830302 PMC6878541

[37] Büssing A Fischer J Haller A Heusser P Ostermann T Matthiessen PF. Validation of the brief multidimensional life satisfaction scale in patients with chronic diseases. Eur J Med Res. (2009) 14:171–7. 10.1186/2047-783X-14-4-171 19380290 PMC3401007

[38] Büssing A Recchia DR Baumann K. The reliance on God's help scale as a measure of religious trust – a summary of findings. Religions. (2015) 6:1358–67. 10.3390/rel6041358

[39] Tingley D Yamamoto T Hirose K Keele L Imai K. mediation: R package for causal mediation analysis. J Stat Soft. (2014) 59:1–38. 10.18637/jss.v059.i05

[40] Hebbali A. olsrr: Tools for Building OLS Regression Models. R Package Version 0.5.3. (2020). Available online at: https://CRAN.R-project.org/package=olsrr (accessed February 22, 2021).

[41] Dahiru T. P - value, a true test of statistical significance? A cautionary note. Ann Ibadan Postgrad Med. (2008) 6:21–6. 10.4314/aipm.v6i1.64038 25161440 PMC4111019

[42] Akoglu H. User's guide correlation coefficients. Turk J Emerg Med. (2018) 18:91–3. 10.1016/j.tjem.2018.08.001 30191186 PMC6107969

[43] Sullivan GM Feinn R. Using effect size-or why the P value is not enough. J Grad Med Educ. (2012) 4:279–82. 10.4300/JGME-D-12-00156.1 23997866 PMC3444174

[44] Luna MJ Ameli R Sinaii N Cheringal J Panahi S Berger A. Gender differences in psycho-social-spiritual healing. J Womens Health. (2019) 28:11. 10.1089/jwh.2019.7837 PMC6862956 31502927

[45] Haidt J Keltner D. Appreciation of beauty and excellence. In: Peterson Cand Seligman MEP, editor. Character Strengths and Virtues. Washington, DC: American Psychological Association (2004). p. 537–51.

[46] Dong R Ni SG. Openness to experience, extraversion, and subjective well-being among chinese college students: the mediating role of dispositional awe. Psychol Rep. (2020) 123:903–28. 10.1177/0033294119826884 30741089

[47] Hayes SC Follette VM Linehan MM. Mindfulness and Acceptance: Expanding the Cognitive-Behavioral Tradition. New York, NY: Guilford Publications (2004).

[48] Kabat-Zinn J. Full Catastrophe Living, Revised Edition: How to Cope With Stress, Pain and Illness Using Mindfulness Meditation. London: Piatkus (2013).

[49] Kabat Zinn J. Mindfulness for Beginners: Reclaiming the Present Moment–And Your Life. Boulder: Sounds True Inc (2016).

